# Analysis of Tear Function Outcomes following Collagen Cross-Linking Treatment in Ectatic Corneas

**DOI:** 10.1155/2022/1910607

**Published:** 2022-03-14

**Authors:** Xiu Wang, Xue Bai, Chengcheng Jin, Liqiong Zhao, Ruihua Wei

**Affiliations:** Tianjin Key Laboratory of Retinal Functions and Diseases, Tianjin Branch of National Clinical Research Center for Ocular Disease, Eye Institute and School of Optometry, Tianjin Medical University Eye Hospital, Fukang Road 251, Nankai District, Tianjin 300384, China

## Abstract

**Purpose:**

To analyze tear function outcomes following collagen cross-linking (CXL) treatment in ectatic corneas.

**Methods:**

Fifty-seven eyes of 34 patients were included, and patients with keratoconus who underwent epithelium-on (epi-on) or epithelium-off (epi-off) CXL were evaluated. The following tests were performed preoperatively and at 1, 3, 6, and 12 months postoperatively: best-corrected visual acuity (BCVA), maximum keratometry value (Kmax), ocular surface disease index (OSDI) questionnaire, slit-lamp examination, tear meniscus height, first noninvasive Keratograph breakup time (1st NIKBUT), average NIKBUT, and bulbar redness.

**Results:**

BCVA improved in both epi-on and epi-off groups at most follow-up points, but was not significantly different between groups. At 12-month follow-up, Kmax in the epi-on and epi-off groups improved after CXL, but there was no significant difference between the groups. The OSDI in both groups decreased after operation compared with before surgery, and there was no significant difference between the two groups. Comparing the two groups, only the change in the tear meniscus height at 6 months postoperatively was statistically significant, and the pre- and postoperative values of the two groups were within the normal range (>0.20 mm). The change was small and had no clinical significance. There was no change in the 1st NIKBUT and average NIKBUT between the epi-on and epi-off groups. A change in bulbar redness was significantly better in the epi-off group than in the epi-on group at 3 months postoperatively. Comparing the effects at 1 year postoperatively, both groups had positive results in OSDI, NIKBUT, tear meniscus height, and bulbar redness.

**Conclusion:**

Both epi-on and epi-off CXL can control the progression of keratoconus, although epi-off CXL is more effective. Both methods have a positive effect on dry eye, which can improve the condition of the tear film and reduce dry eye symptoms in patients with keratoconus.

## 1. Introduction

Keratoconus is a progressive primary ectatic disease, which involves irregular astigmatism and corneal thinning due to structural and biomechanical changes in the stroma, leading to vision loss [1, 2]. Keratoconus frequently begins in puberty and progresses until the age of 30–40 years [3]. It can lead to irreversible vision loss and requires treatment via keratoplasty. Treatment is selected based on the severity of the disease and is aimed at improving vision and preventing ectasia progression [2]. Corneal collagen cross-linking (CXL) is a surgical treatment used to stabilize corneal ectasia and increase corneal strength [4]. Various CXL protocols have been extensively investigated and applied [5], including epithelium-on (epi-on) and epithelium-off (epi-off) protocols with transepithelial riboflavin application. Both of these surgical methods are popular and well researched [6, 7]. Although keratoconus is associated with dry eye [8], alterations in tear indices after any corneal procedure are of concern to the surgeon. Previous studies mainly reported the effects on the ocular surface in keratoconus via epi-off and did not examine epi-on CXL or compare the two kinds of surgery [3, 9]. Furthermore, all studies evaluated Caucasian patients. A comparison of the effects of the two surgical methods on tear film function is important for the choice of surgical method. In our study, we analyzed the influence of epi-on and epi-off CXL on the tear function of Chinese patients with keratoconus.

## 2. Materials and Methods

### 2.1. Patients

This prospective study protocol was approved by the Institutional Review Board of Tianjin Medical University Eye Hospital (Tianjin, China). The clinical study registration number was ChiCTR2000032444. Patients or their parents provided written informed consent for study procedures and protocols, which complied with the principles of the Declaration of Helsinki.

Keratoconus frequently begins in puberty and progresses until the age of 30–40 years. Due to the influence of age on tear film function, this study only included patients <40 years of age. Patients were prospectively recruited; 57 keratoconic eyes of 34 patients from the Tianjin Medical University Eye Hospital (Tianjin, China) between May 2020 and September 2020 were included. Of the 57 keratoconic eyes, 26 were included in the epi-on group and 31 in the epi-off group.

Patients were excluded if they had a history of systemic disease, any ocular autoimmune disease, keratitis, glaucoma, ocular injury or surgery, any other ocular surface disease, such as meibomian gland dysfunction, or previous treatment with systemic steroids or topical medication for artificial tears.

### 2.2. Treatments

Progression of keratoconus was defined as a 1.00-diopter (*D*) corneal change in the maximum keratometry value (Kmax) and a 20-*μ*m reduction in the central corneal thickness, which were determined using a Pentacam Scheimpflug tomography analyzer (Nidek Co, Ltd., Gamagori, Japan) [10]. CXL was performed using an epi-on or epi-off protocol for the eyes. At 6 weeks after CXL, all patients underwent fittings for Rose-K rigid gas permeable (RGP) contact lenses (Rose-K RGPs, FreshKon, Shanghai, China), which were performed by a professional team.

### 2.3. Data Collection

At each visit, data were collected regarding slit-lamp examination findings, best-corrected visual acuity (BCVA) using a conventional Snellen chart and described in the decimal visual acuity style, and Kmax value in the 3-mm diameter central zone, which were measured using corneal topography (Orbscan IIz; Bausch & Lomb Surgical). Each patient was asked to complete an ocular surface disease index (OSDI) questionnaire, which is reliable and valid for evaluating the severity of dry eye disease [11, 12]. The OSDI questionnaire was assessed based on a 0–100 scale, with higher scores representing more severe disease and greater disability [11]. In addition, we used the Keratograph 5M to evaluate the first noninvasive Keratograph breakup time (1st NIKBUT), the average NIKBUT, tear meniscus height, and bulbar redness. The averages were obtained from three measurements of the 1st and average NIKBUT and the tear meniscus height [13].

The examinations were performed chronologically by the same ophthalmologist. Tests were performed for the right eye and then the left eye preoperatively and at 1, 3, 6, and 12 months postoperatively. All visits were conducted in the morning, between 10 and 12 AM. The patient stopped wearing RGP contact lenses for 1 week before each visit to avoid the influence of RGP on the ocular surface. The incidence of complications was recorded at the 12-month follow-up.

### 2.4. Surgical Procedures for Epi-On and Epi-Off CXL

The epi-on CXL procedure began by measuring the corneal thickness to ensure that it was >400 *μ*m. Enough ParaCel solution (Vibex Rapid; Avedro Inc., Waltham, MA, USA) was applied to completely cover the cornea, and this process was repeated every 90 s for 4 min. The cornea was fully flushed using VibeX Xtra (Vibex Rapid; Avedro Inc, Waltham, MA, USA), which was then applied to completely cover the cornea; this process was repeated every 90 s for 6 min. Ultraviolet light treatment was commenced using the KXL System, after which the procedure was completed using standard techniques.

The epi-off CXL procedure was commenced by removing the corneal epithelium over the desired area. A sufficient amount of riboflavin ophthalmic solution (Vibex Rapid; Avedro Inc., Waltham, MA, USA) was applied to completely cover the exposed stroma. This procedure was repeated at 2-min intervals up to a total of 30 min based on the desired depth of cross-linking. The corneal thickness was subsequently measured to ensure that it was >400 *μ*m; thereafter, riboflavin ophthalmic solution was applied to completely cover the exposed stroma. Ultraviolet light treatment was commenced using the KXL cross-linking device (Avedro Inc., Waltham, MA, USA). The procedure was then completed using standard techniques.

A bandage contact lens was applied for the patients after the procedure. During the postoperative period, patients were instructed to use fluorometholone eye drops (Santen Inc., Osaka, Japan) 4 times daily for 1 week after the procedure. The eye drop dosage was tapered over the following 4 weeks. Patients were also instructed to use levofloxacin eye drops (Santen Inc., Osaka, Japan) for the first 3 days after the procedure. The bandage contact lens was removed on postoperative day 3.

### 2.5. Statistical Analysis

Statistical analyses were performed using SPSS (version 26.0; IBM Corp., Armonk, NY, USA). All data are expressed as mean ± standard deviation. Normal data distribution was tested using the one-sample Kolmogorov–Smirnov test. Changes between different groups were analyzed using independent samples *t*-test (tear meniscus height and bulbar redness) and Mann–Whitney *U* test (BCVA, OSDI, 1st NIKBUT, average NIKBUT). The generalized estimating equation was used to compare outcomes preoperatively and at 1, 3, 6, and 12 months postoperatively. This equation was calculated to account for intereye correlation for eye-specific measurements. The lower and upper bounds of the 95% confidence intervals (95% CI) are shown in Tables 1–3. The relationship between the different parameters was assessed using Spearman's correlation analysis. *P* < 0.05 was considered statistically significant.

## 3. Results

The demographic characteristics and baseline findings of the patients are shown in Table 4. We evaluated 57 keratoconic eyes in 34 patients (24 males, 10 females). The mean ages of the epi-on and epi-off groups were 25.46 ± 4.90 and 22.77 ± 6.09 years (*P* < 0.076), respectively. There was no significant difference between the two groups with regard to BCVA, OSDI, tear meniscus height, 1st NIKBUT, average NIKBUT, or bulbar redness before surgery (*P* > 0.05).No patient experienced treatment-related adverse events, and all patients initiated wearing their RGP lenses at 6 weeks postoperatively without any sign of intolerance. During the study period, no infectious keratitis was observed. No patient dropped out during the study.

A highly significant inverse correlation was observed between the value of Kmax and BCVA in the two groups (*r* = −0.772, *P* < 0.01) (Table 5). A positive correlation was found between 1st NIKBUT and average NIKBUT in the two groups (*r* = 0.696, *P* < 0.01).

**Figure 1:**
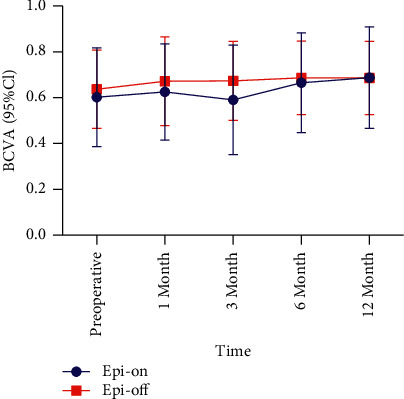
Comparison of the BCVA during the 12-month follow-up period. BCVA: Best-corrected visual acuity; CI: confidence interval.

The BCVA values in the epi-on group were higher than those preoperatively at 1, 6, and 12 months postoperatively (*P* > 0.05) (Table 1, Figure 1). The BCVA values at each follow-up point in the epi-off group were higher than those before surgery (*P* > 0.05) (Table 2, Figure 1). There was no significant difference in pre- and postoperative BCVA between the groups (*P* > 0.05) (Table 3).

**Figure 2:**
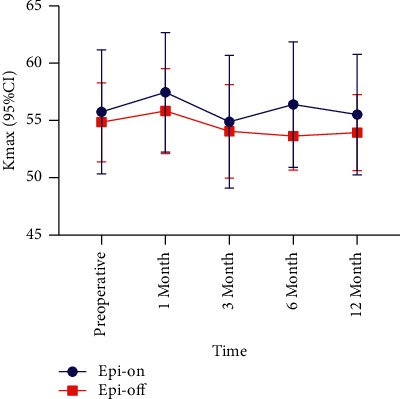
Comparison of Kmax during the 12-month follow-up period. Kmax: Maximum keratometry; CI: confidence interval.

In the epi-on group, Kmax decreased at 3 (△ = −0.86, *P*=0.028) and 12 (△ = −0.24, *P*=0.293) months postoperatively, while at 1 (△ = 1.71, *P*=0.533) and 6 months (△ = 0.65, *P*=0.789) postoperatively, Kmax was steeper than at preoperatively (Tables 1 and 3, Figure 2). In the epi-off group, there were steeper Kmax values at 1 month postoperatively (△ = 0.98, *P*=0.294) (Tables 2 and 3, Figure 2). Kmax values also decreased in the epi-off group, with significant differences between preoperative and postoperative values at 6 months (△ = −1.20, *P*=0.048). And Kmax also decreased at 12 (△ = −0.91, *P*=0.105) months postoperatively. However, there was no significant difference in pre- and postoperative Kmax values between the groups at each follow-up points (*P* > 0.05).

**Figure 3:**
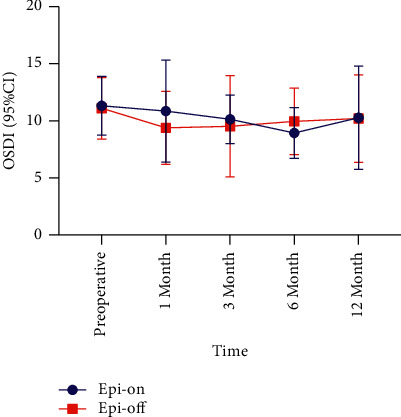
Comparison of the OSDI scores during the 12-month follow-up period. OSDI: Ocular surface disease index; CI: confidence interval.

In the epi-off group, the OSDI at the 12-month follow-up after surgery was significantly lower than that before surgery (△ = −0.90, *P*=0.048) (Tables 2 and 3, Figure 3). The other follow-up measures of the two groups also reduced to varying degrees compared with the preoperative values (*P* > 0.05) (Tables 1–3).

**Figure 4:**
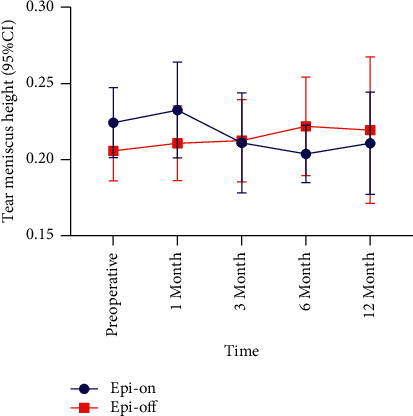
Comparison of the tear meniscus height during the 12-month follow-up period. CI: confidence interval.

There was a slight change in the tear meniscus height between the two groups at each follow-up compared with that before surgery (Tables 1–3), Figure 4). Only the epi-off group (△ = 0.02, *P*=0.331) at the 6-month follow-up was significantly different (*P*=0.036) from the epi-on group (△ = −0.02, *P*=0.151).

**Figure 5:**
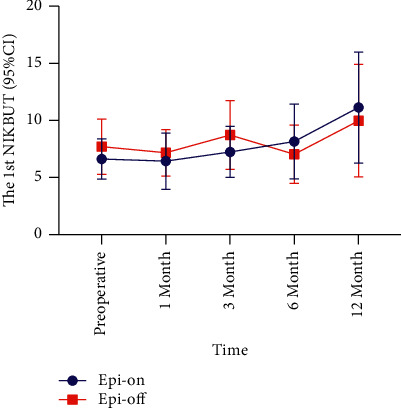
Comparison of the 1st NIKBUT during the 12-month follow-up period. NIKBUT: Noninvasive Keratograph breakup time; CI: confidence interval.

The 1st NIKBUT of the epi-on group increased gradually from the postoperative third month compared with that preoperatively, and there was a significant difference between postoperative 12 months and preoperatively (△ = 5.73, *P*=0.001) (Tables 1 and 3, Figure 5). The 1st NIKBUT in the epi-off group decreased at 1 (△ = −0.55, *P* = 0.939) and 6 months (△ = −0.67, *P*=0.726) postoperatively compared with the preoperative values, while it increased at 3 (△ = 1.02, *P*=0.953) and 12 months (△ = 2.26, *P*=0.099) postoperatively compared with the preoperative values (Tables 2 and 3). There was no significant difference in 1st NIKBUT between the two groups at each follow-up point (*P* > 0.05) (Table 3).

**Figure 6:**
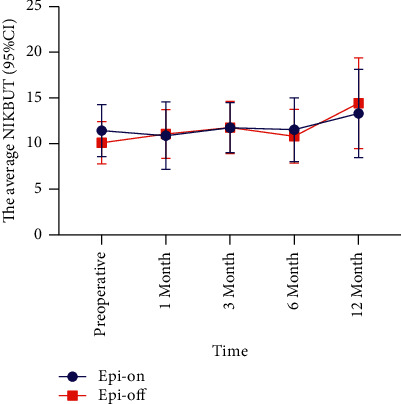
Comparison of the average NIKBUT during the 12-month follow-up period. NIKBUT: Noninvasive Keratograph breakup time; CI: confidence interval.

The average NIKBUT of the epi-on group decreased at 1 month postoperatively (△ = −0.55, *P*=0.021) and was statistically significant, and then gradually increased from the third month onward (*P* > 0.05) (Tables 1 and 3, Figure 6). The average NIKBUT of the epi-off group increased after surgery compared with that before surgery, and there was a significant difference between the 12-month post- and preoperative values (△ = 4.34, *P* < 0.01) (Tables 2 and 3).

**Figure 7:**
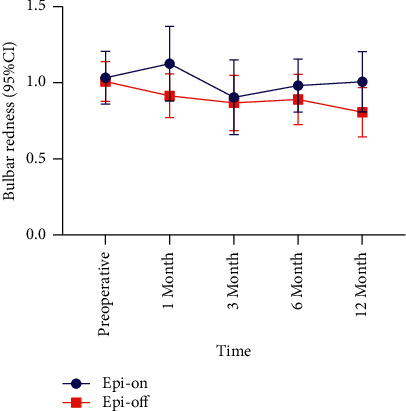
Comparison of the bulbar redness during the 12-month follow-up period. CI: confidence interval.

The bulbar redness of the epi-on group decreased at 6 (△ = −0.05, *P*=0.289) and 12 months (△ = −0.02, *P*=0.426) postoperatively and increased at other time points compared with that of the preoperative period (*P* > 0.05) (Tables 1 and 3, Figure 7). The bulbar redness of the epi-off group was lower at each follow-up than it was preoperatively, and there was a significant difference between score values preoperatively and at 12 months postoperatively (△ = −0.20, *P*=0.001) (Tables 2 and 3, Figure 7). There was a significant difference in the pre- and postoperative bulbar redness between the groups at 3 months postoperatively (*P*=0.021) (Table 3).

## 4. Discussion

The standard technique for corneal stabilization in cases of keratoconus is CXL, which aims at increasing biomechanical stromal stability and stopping disease progression via the formation of new chemical bonds between collagen fibrils and extracellular matrix proteins in the stroma [14–16]. It is well known that keratoconus is associated with dry eye [8, 17]. Considering the ocular morbidity that dry eye can cause, therapeutic approaches to managing keratoconus should not worsen any underlying dry eye. Few studies have compared epi-on and epi-off surgery in terms of visual and tear film functional changes, or even in terms of the progression of keratoconus [18, 19]. Therefore, we studied the effects of two surgical methods on BCVA, which are described as decimal visual acuity style and tear film function of patients with keratoconus. The Tear Film and Ocular Surface Society Dry Eye Workshop suggested that a noninvasive measure of tear stability was preferred in the diagnosis of dry eye [20, 21]. We assessed the tear function of patients with keratoconus using Keratograph 5M to provide an effective NIKBUT, tear meniscus height, and bulbar redness score.

In our study, the BCVA of the epi-on and epi-off groups increased at most follow-up time points, indicating that both epi-on and epi-off CXL are beneficial to vision improvement. BCVA and Kmax also had a negative correlation; therefore, improvement in visual acuity after CXL was consistent with the decrease in Kmax in the two groups. However, this conclusion did not apply to the changes of BCVA and Kmax in the epi-on and epi-off groups at 1 and 6 months postoperatively in the epi-on group. Kmax increased at 1 month after epi-on and epi-off CXL, but BCVA also increased. In Weijun Jian's study, the Kmax values were 60.10 ± 7.51 *D* preoperatively and 61.42 ± 8.92, 61.17 ± 7.96, and 60.02 ± 7.58 *D* at 1, 6, and 12 months after accelerated transepithelial CXL (*P*=0.05), respectively [22]. Kmax in the early postoperative period was also higher than that before surgery. The specific mechanism for this has not yet been reported. Caporossi et al. evaluated 26 eyes of 26 patients treated with epi-on CXL. They found that visual acuity had improved up to 6 months, stabilized at 12 months, and worsened at 24 months. They concluded that epi-on CXL was not sufficiently effective to stop the progression of keratoconus in pediatric patients [23]. In our study, the epi-off group also showed greater reduction in Kmax than the epi-on group at 12 months. Therefore, the impact of epi-on CXL on BCVA and Kmax needs to be explored with a larger number of patients and longer follow-up time.

The cornea is one of the most densely innervated tissues in the human body. Corneal nerve damage caused by trauma, disease, or surgery can reduce corneal sensitivity and affect the functional integrity of the ocular surface [24]. The differences in the time course of neural rehabilitation between different surgeries may be due to differences in both the cause and extent of the initial neural injury [25]. The removal of the corneal epithelium and ultraviolet A exposure during CXL can cause damage to the subepithelial nerve plexus, which can cause decreased corneal sensitivity [26]. Our results revealed that all patients, regardless of the treatment plan, had a relatively stable OSDI at each follow-up (*P*  >  0.05). This result is consistent with that of a previous study [3]. The stability of OSDI in the early stage after surgery—that is, the reduction of subjective dry eye symptoms—is related to corneal nerve damage. With the repair of corneal nerves and improvement in corneal sensitivity postoperatively, the stability of the OSDI is related to the recovery of tear film stability. Thus, CXL had no effect on the patient's dry eye perception, and the postoperative dry eye symptoms did not affect the patient's quality of life. Among patients enrolled in our study, the noninvasive assessments did not correlate with the severity of symptoms of the OSDI. This conclusion is consistent with that of the study by Sutphin et al. [13].

Loss of corneal sensation is expected to adversely affect blinking and basic tear secretion. There was no significant difference in our study in tear meniscus height pre- and postoperatively, indicating that CXL had no effect on tear secretion [27]. The sensitivity of the peripheral unaffected part of the cornea during the procedure may be sufficient to regulate basic tear secretion, or the aberrant activity of the amputated corneal nerves may contribute to this [28].

The 1st NIKBUT in the epi-on group was significantly increased 12 months postoperatively than that before surgery. Moreover, the average NIKBUT in the epi-off group was significantly increased at 12 months postoperatively when compared to its preoperative values. The Kmax values of the two groups also decreased at 12 months postoperatively than that before surgery. Dogru et al. [29] demonstrated the relationship between tear film stability and corneal curvature changes in patients with keratoconus. They found that tear film breakup time (TBUT) values were significantly lower in moderate and severe keratoconic eyes compared to mild keratoconus. In the study by Mazzotta et al., the CXL-induced corneal flattening, the epithelial progressive stratification regularizing the symmetry of the corneal surface, may lead to a smoother corneal surface [30]. They also demonstrated a healthier corneal epithelium formed after CXL, which may result in better quality and a higher quantity of tear mucin, which could explain the stable TBUT levels. This conclusion was the same as ours; that is, CXL can increase tear film stability. In previous studies, epi-off CXL had better Kmax values [31]. Therefore, postoperative flat cornea is conducive to the coating of the tear film. In our study, the changes in OSDI, average NIKBUT, and bulbar redness were more obvious in the epi-off group preoperatively and at 12 months postoperatively. Uysal et al. also demonstrated improvement in TBUT 18 months after CXL in keratoconic eyes, which they attributed to reduced corneal irregularity after CXL [32].

The bulbar redness scores were unchanged in the epi-on group but significantly decreased in the epi-off group at 12 months postoperatively compared with that before surgery (*P*=0.001). In Uysal's study [32], temporal conjunctival squamous metaplasia and goblet cell density improved together with increased TBUT 18 months after CXL. The exposed conjunctiva is more meaningful in terms of dry eye syndrome [33], and the increased tear film stability may provide a more humid ocular surface and subsequent improvement in bulbar redness. This suggests that CXL did not cause additional inflammation. Moreover, epi-off CXL has a more positive effect in reducing bulbar redness scores.

Our study suggests that both epi-on and epi-off CXL can control the progression of keratoconus, and epi-off CXL is more effective. Both epi-on and epi-off have a positive effect on dry eye, which can improve the condition of the ocular surface and reduce the symptoms of dry eye in patients.

Cessation of RGP use for more than 1 week will significantly affect the patient's visual quality and quality of life. Therefore, in this study, the patient only stopped wearing RGP for 1 week. Future studies can add a control group, wherein patients with keratoconus will not undergo surgery, but the changes in tear film function before and after wearing RGP alone will be compared. In our study, only the OSDI questionnaire, tear meniscus height, 1st NIKBUT, average NIKBUT, and bulbar redness were included. Other tear film parameters, such as tear osmolarity, lid wiper epitheliopathy, tear clearance, and fluorescein staining, would have provided a broader perspective to the study. In addition, the inclusion of both eyes of the same patient in our study may cause potential statistical bias. Finally, the findings of the present study are limited by the small sample size. Thus, a large sample size study with monocular inclusion of study subjects and different age groups is required to confirm how CXL affects the ocular surface in patients with keratoconus, which may guide the selection of individualized treatment plans.

## Figures and Tables

**Table 1:** Analysis of parameter changes in the epi-on group during the follow-up period.

Parameter	Preoperative	1 month	3 months	6 months	12 months
BCVA	0.60 ± 0.36 (0.24–0.90)	0.63 ± 0.33 (0.49–0.98)	0.59 ± 0.33 (0.34–1.06)	0.66 ± 0.36 (0.33–1.08)	0.69 ± 0.37 (0.36–1.12)
*P* value		0.436	0.832	0.303	0.090
Kmax (D)	55.75 ± 8.95 (45.83–68.56)	57.46 ± 7.78 (47.70–66.80)	54.89 ± 8.63 (45.43–66.61)	56.40 ± 8.62 (45.89–68.48)	55.52 ± 8.70 (46.02–48.65)
*P* value		0.533	0.028^*∗*^	0.789	0.293
OSDI (score)	11.33 ± 6.10 (8.76–13.91)	10.86 ± 7.74 (6.39–15.33)	10.14 ± 4.78 (8.02–12.26)	8.94 ± 4.45 (6.73–11.16)	10.27 ± 6.72 (5.76–14.79)
*P* value		0839	0.450	0.434	0.908
Tear meniscus height (mm)	0.22 ± 0.05 (0.20–0.25)	0.23 ± 0.06 (0.20–0.26)	0.21 ± 0.07 (0.18–0.24)	0.20 ± 0.04 (0.18–0.22)	0.21 ± 0.06 (0.18–0.24)
*P* value		0.630	0.694	0.151	0.240
1st NIKBUT (s)	6.62 ± 3.65 (4.86–8.39)	6.43 ± 4.06 (3.98–8.89)	7.25 ± 4.33 (5.02–9.48)	8.16 ± 6.57 (4.90–11.43)	11.14 ± 7.64 (6.28–15.99)
*P* value		0.181	0.164	0.067	0.001^*∗*^
Average NIKBUT (s)	11.42 ± 5.93 (8.56–14.28)	10.87 ± 6.11 (7.18–14.57)	11.75 ± 5.32 (9.02–14.48)	11.53 ± 7.02 (8.03–15.02)	13.30 ± 7.61 (8.47–18.14)
*P* value		0.021^*∗*^	0.971	0.637	0.146
Bulbar redness (score)	1.03 ± 0.41 (0.86–1.21)	1.13 ± 0.46 (0.88–1.37)	1.05 ± 0.43 (0.85–1.24)	0.98 ± 0.39 (0.81–1.16)	1.01 ± 0.34 (0.81–1.21)
*P* value		0.549	0.711	0.289	0.426

BCVA: Best-corrected visual acuity (decimal visual acuity style); Kmax: maximum keratometry; OSDI: ocular surface disease index; NIKBUT: Noninvasive Keratograph breakup time; SD: standard deviation. ^*∗*^*P* value means that the test level has a statistical difference at 0.05, and ^*∗∗*^*P* value means that the test level has a statistical difference at 0.01. “()” means 95% CI (CI is confidence interval).

**Table 2:** Analysis of parameter changes in the epi-off group during the follow-up period.

Parameter	Preoperative	1 month	3 months	6 months	12 months
BCVA	0.64 ± 0.31 (0.29–0.86)	0.67 ± 0.30 (0.40–0.94)	0.67 ± 0.28 (0.45–0.95)	0.69 ± 0.29 (0.46–0.97)	0.69 ± 0.28 (0.45–0.95)
*P* value		0.111	0.107	0.290	0.111
Kmax (D)	54.85 ± 6.22 (50.71–63.26)	55.83 ± 6.14 (52.24–63.85)	54.05 ± 6.07 (50.55–61.37)	53.65 ± 5.38 (50.41–61.22)	53.95 ± 6.00 (50.39–63.04)
*P* value		0.294	0.306	0.048^*∗*^	0.105
OSDI	11.10 ± 7.19 (8.42–13.78)	9.39 ± 7.41 (6.19–12.60)	9.53 ± 9.22 (5.08–13.97)	9.96 ± 6.75 (7.04–12.88)	10.20 ± 6.92 (6.37–14.03)
*P* value		0.515	0.195	0.336	0.048^*∗*^
Tear meniscus height (mm)	0.21 ± 0.05 (0.19–0.23)	0.21 ± 0.06 (0.19–0.24)	0.21 ± 0.07 (0.19–0.24)	0.22 ± 0.07 (0.19–0.25)	0.22 ± 0.09 (0.17–0.27)
*P* value		0.631	0.810	0.331	0.837
1st NIKBUT (s)	7.71 ± 5.59 (5.29–10.13)	7.17 ± 4.49 (5.12–9.21)	8.73 ± 6.43 (5.72–11.74)	7.05 ± 4.95 (4.50–9.59)	9.98 ± 7.75 (5.05–14.90)
*P* value		0.939	0.953	0.726	0.099
Average NIKBUT (s)	10.09 ± 5.36 (7.77–12.40)	11.06 ± 5.86 (8.39–13.73)	11.77 ± 6.62 (8.67–14.86)	10.81 ± 5.73 (7.86–13.75)	14.43 ± 7.81 (9.47–19.39)
*P* value		0.091	0.232	0.206	<0.01^*∗∗*^
Bulbar Redness (score)	1.01 ± 0.36 (0.88–1.14)	0.91 ± 0.37 (0.77–1.06)	0.87 ± 0.44 (0.69–1.05)	0.89 ± 0.37 (0.73–1.06)	0.81 ± 0.29 (0.65–0.97)
*P* value		0.569	0.556	0.230	0.001^*∗∗*^

BCVA: Best-corrected visual acuity (decimal visual acuity style); Kmax: maximum keratometry; OSDI: ocular surface disease index; NIKBUT: Noninvasive Keratograph breakup time; SD: standard deviation. ^*∗*^*P* value means that the test level has a statistical difference at 0.05, and ^*∗∗*^*P* value means that the test level has a statistical difference at 0.01. “()” means 95% CI (CI is confidence interval).

**Table 3:** Analysis of parameter changes between the two groups.

Variable	Group	Δ1 Month mean ± SD	Δ3 Months mean ± SD	Δ6 Months mean ± SD	Δ12 Months mean ± SD
BCVA	Epi-on	0.02 ± 0.49 (−0.12–0.45)	−0.01 ± 0.49 (−0.13–0.40)	0.06 ± 0.51 (−0.11–0.38)	0.09 ± 0.52 (−0.04–0.37)
	Epi-off	0.04 ± 0.44 (−0.04–0.24)	0.04 ± 0.42 (−0.05–0.31)	0.05 ± 0.43 (−0.03–0.32)	0.05 ± 0.42 (−0.04–0.29)
*P* value		0.652	0.525	0.899	0.767
Kmax (D)	Epi-on	1.71 ± 11.89 (−2.74–2.84)	−0.86 ± 12.43 (−2.39–0.02)	0.65 ± 12.43 (−0.45–0.42)	−0.24 ± 12.49 (−0.29–0.56)
	Epi-off	0.98 ± 8.74 (−0.19–2.30)	−0.80 ± 8.69 (−3.19–1.00)	−1.20 ± 8.21 (−2.97–0.62)	−0.91 ± 8.64 (−1.57–1.03)
*P* value		0.576	0.818	0.213	0.406
OSDI (score)	Epi-on	−0.48 ± 9.91 (−3.74–5.46)	−1.20 ± 7.68 (−4.93–1.57)	−2.39 ± 7.43 (−6.07–0.85)	−1.06 ± 9.15 (−4.00–3.09)
	Epi-off	−1.71 ± 10.46 (6.64–0.57)	−1.57 ± 12.01 (−8.05–0.26)	−1.14 ± 9.87 (−1.58–2.27)	−0.90 ± 9.99 (−2.55–1.88)
*P* value		0.112	0.107	0.254	0.896
Tear meniscus height (mm)	Epi-on	0.01 ± 0.08 (−0.01–0.05)	−0.01 ± 0.09 (−0.05–0.02)	−0.02 ± 0.07 (−0.04–0.00)	−0.01 ± 0.08 (−0.05–0.03)
	Epi-off	0.00 ± 0.08 (−0.02–0.02)	0.01 ± 0.08 (−0.02–0.02)	0.02 ± 0.09 (−0.01–0.04)	0.01 ± 0.10 (−0.03–0.05)
*P* value		0.226	0.208	0.036^*∗*^	0.615
1st NIKBUT (s)	Epi-on	−0.19 ± 5.47 (−3.22–2.54)	0.62 ± 5.64 (−1.67–2.98)	1.54 ± 7.21 (−1.87–5.57)	4.51 ± 7.82 (2.07–9.39)
	Epi-off	−0.55 ± 7.13 (−4.26–2.81)	1.02 ± 8.50 (−3.38–5.31)	−0.67 ± 7.45 (−4.95–3.05)	2.26 ± 9.40 (−1.64–3.85)
*P* value		0.972	0.976	0.488	0.066
Average NIKBUT (s)	Epi-on	−0.55 ± 8.53 (−5.17–2.29)	0.32 ± 7.98 (−2.48–2.68)	0.10 ± 9.19 (−3.58–4.35)	1.88 ± 9.55 (0.35–8.27)
	Epi-off	0.97 ± 7.93 (−2.32–3.94)	1.68 ± 8.45 (−2.14–6.38)	0.72 ± 7.83 (−4.25–4.62)	4.34 ± 9.30 (−1.51–7.09)
*P* value		0.357	0.345	1.000	0.759
Bulbar redness (score)	Epi-on	0.09 ± 0.61 (−0.09–0.19)	0.01 ± 0.59 (−0.04–0.20)	−0.05 ± 0.57 (−0.28–0.09)	−0.02 ± 0.53 (−0.24–0.24)
	Epi-off	−0.09 ± 0.52(−0.21–0.10)	−0.14 ± 0.58 (−0.26–0.05)	−0.12 ± 0.52 (−0.34–0.04)	−0.20 ± 0.46 (−0.63–0.12)
*P* value		0.721	0.021^*∗*^	0.628	0.062

BCVA: Best-corrected visual acuity (decimal visual acuity style); Kmax: maximum keratometry; OSDI: ocular surface disease index; NIKBUT: Noninvasive Keratograph breakup time; SD: standard deviation. “Δ” means the difference between pre- and postoperative values.

**Table 4:** Demographic characteristics and baseline evaluation.

Characteristics	Epi-on	Epi-off	*P* value
Eyes	26	31	
Age (years)			
Mean ± SD	25.46 ± 4.90	22.77 ± 6.09	0.076
Range	14–33	15–32	
BCVA	0.60 ± 0.36	0.64 ± 0.31	0.537
Kmax (D)	55.75 ± 8.95	54.85 ± 6.22	0.942
OSDI (score)	11.33 ± 6.10	11.10 ± 7.19	0.773
Tear meniscus height (mm)	0.22 ± 0.05	0.21 ± 0.05	0.336
1st NIKBUT (s)	6.62 ± 3.65	7.71 ± 5.59	0.771
Average NIKBUT (s)	11.42 ± 5.93	10.09 ± 5.36	0.587
Bulbar redness (score)	1.03 ± 0.41	1.01 ± 0.36	0.912

BCVA: Best-corrected visual acuity (decimal visual acuity style); Kmax: maximum keratometry; OSDI: ocular surface disease index; NIKBUT: Noninvasive Keratograph breakup time; SD: standard deviation.

**Table 5:** Correlation among ocular parameters.

	BCVA	Kmax (D)	OSDI (score)	Tear meniscus height (mm)	1st NIKBUT (s)	Average NIKBUT (s)	Bulbar redness (score)
BCVA	1.000	−0.772^*∗∗*^	−0.152	0.149	−0.58	−0.081	0.023
Kmax (D)		1.000	0.152	−0.280	0.242	0.169	−0.088
OSDI (score)			1.000	0.104	−0.022	0.157	−0.101
Tear meniscus height (mm)				1.000	0.197	0.233	−0.191
1st NIKBUT (s)					1.000	0.696^*∗∗*^	−0.049
Average NIKBUT (s)						1.000	0.190
Bulbar Redness (score)							1.000

BCVA: Best-corrected visual acuity (decimal visual acuity style); Kmax: maximum keratometry; OSDI: ocular surface disease index; NIKBUT: noninvasive Keratograph breakup time. ^*∗*^*P* value means that the test level has a significant difference at 0.05, and ^*∗∗*^*P* value means that the test level has a significant difference at 0.01.

## Data Availability

The data used to support the findings of this study are available from the corresponding author upon request.
